# A New SDM-Based Approach for Assessing Climate Change Effects on Plant–Pollinator Networks

**DOI:** 10.3390/insects15110842

**Published:** 2024-10-28

**Authors:** Ehsan Rahimi, Chuleui Jung

**Affiliations:** 1 Agricultural Science and Technology Institute, Andong National University, Andong 36729, Republic of Korea; ehsanrahimi666@gmail.com; 2 Department of Plant Medical, Andong National University, Andong 36729, Republic of Korea

**Keywords:** species distribution models, rewiring, mutualistic networks, network extraction, Chile

## Abstract

Current methods for studying the effects of climate change on plants and pollinators can be grouped into two main categories. The first category involves using species distribution models (SDMs) to generate habitat suitability maps, followed by applying climate change scenarios to predict the future distribution of plants and pollinators separately. The second category involves constructing interaction matrices between plants and pollinators and then either randomly removing species or selectively removing generalist or specialist species, as a way to estimate how climate change might affect the plant–pollinator network. The primary limitation of the first approach is that it examines plant and pollinator distributions separately, without considering their interactions within the context of a pollination network. The main weakness of the second approach is that it does not accurately predict climate change impacts, as it arbitrarily selects species to remove without knowing which species will truly shift, decline, or increase in distribution due to climate change. Therefore, a new approach is needed to bridge the gap between these two methods while avoiding their specific limitations. In this context, we introduced an innovative approach that first requires the creation of binary climate suitability maps for plants and pollinators, based on SDMs, for both the current and future periods. This step aligns with the first category of methods mentioned earlier. To assess the effects of climate change within a network framework, we consider species co-overlapping in a geographic matrix. For this purpose, we developed a Python program that overlays the binary distribution maps of plants and pollinators, generating interaction matrices. These matrices represent potential plant–pollinator interactions, with a ‘0’ indicating no overlap and a ‘1’ where both species coincide in the same cell. As a result, for each cell within the study area, we can construct interaction matrices for both the present and future periods. This means that for each cell, we can analyze at least two pollination networks based on species co-overlap. By comparing the topology of these matrices over time, we can infer how climate change might affect plant–pollinator interactions at a fine spatial scale. We applied our methodology to Chile as a case study, generating climate suitability maps for 187 plant species and 171 pollinator species, resulting in 2906 pollination networks. We then evaluated how climate change could affect the network topology across Chile on a cell-by-cell basis. Our findings indicated that the primary effect of climate change on pollination networks is likely to manifest more significantly through network extinctions, rather than major changes in network topology.

## Simple Summary

Current methods for studying climate change impacts on plants and pollinators fall into two categories: one uses species distribution models (SDMs) to create habitat suitability maps, while the other constructs interaction matrices to estimate changes in plant–pollinator networks by removing species. The first approach is limited as it analyzes species separately, without accounting for the effects of climate change within networks. The second lacks accuracy due to the arbitrary species removal without understanding actual distribution shifts. To address these gaps, we introduced an innovative method that involves generating binary climate suitability maps and assessing species co-overlapping in a geographic matrix to understand potential interactions.

## 1. Introduction

Plant–pollinator interactions play a key functional role in ecosystems because they both facilitate the reproduction of plant species across generations and also provide direct and indirect opportunities for animal feeding [1,2]. New calculations indicate that 90% of flowering plant species are animal-pollinated [3], and up to 90% of tree species in the tropics depend on interactions with animals to complete their life cycles, either through the pollination of flowers or dispersal of seeds [4]. Globally, 74% of crops that rely on animal pollinators are highly dependent on them, with over 40% of their total production being linked to animal pollination [5]. Additionally, at least 35% of the world’s food products are directly dependent on pollinators, and therefore, pollinators are of considerable economic importance. However, these mainly mutualistic interactions could be disrupted by a range of factors including climate change [1], land use alteration, changes in agriculture practices [6,7], lack of flower diversity, and increasing pests and pathogens [8]. Likely, such factors are also threatening the pollination services provided [9]. 

In the natural world, plants and pollinators are interconnected, reliant on multiple other species for their survival. These intricate relationships create complex webs where the disappearance of a single species can trigger significant consequences, directly and indirectly affecting other species within their community [10]. This chain reaction of extinction can set off a domino effect, reshaping how the community operates and disrupting essential functions within the ecosystem [11]. Although plant–pollinator relationships have developed throughout the evolutionary process, climate changes are happening at a more rapid pace, and the plants and pollinators cannot adapt to these changes [12,13,14]. A synthesis of 688 published papers showed that species interactions may be more susceptible to global environmental change than species themselves and that there are clear patterns in the way in which these interactions are eroded [10,15]. 

The direct effects of climate change on pollinating insects include stimulating or limiting the activity of larvae or adults, their distribution, phenology, and growth time length. The indirect effects include the impact on host plant phenology, food quality, predators, and parasites [16]. One of the most important effects of climate change on plant–pollinator interactions is temporal and spatial mismatches [17,18]. It is widely accepted that climate change can affect the geographic ranges of species [19]. Perhaps some of the most critical yet understudied factors affecting the decline in global pollinator populations and pollination services are the alterations in local and regional climatic conditions [20,21]. Indeed, early signs of climate change may be identified by species range shifts [22,23].

Plant–pollinator interactions can be disrupted in at least two ways: through temporal (phenological) and spatial (distributional) mismatches that may change the availability of mutualistic partners [1]. Mismatches in the interactions between plants and pollinators will lead to the emergence of new mutualistic networks [1,24,25]. Though some networks will experience the loss of existing interactions, others could gain novel interactions. In this context, “forbidden links” refer to connections between plants and pollinators that are impossible due to their spatial distributions not overlapping [26,27]. This includes scenarios such as a canopy-dwelling plant and a ground-foraging pollinator existing in separate spaces, or even spatial disparities in ranges occurring on much broader geographic scales [9]. Any changes in temporal and spatial overlap in the future distribution of species due to climate change and other unknown factors may lead to the formation of forbidden links and can drastically change an observed network structure [28].

A few studies have considered spatial mismatches in plant and pollinator communities at the same time or have observed this phenomenon in the field. Since the occurrence of interaction requires the presence of the two species, if a plant species and the animals that pollinate it occupy slightly different climatic niches, a warming climate has the potential to cause a spatial mismatch between the two if their distributions diverge [13]. The potential effects of climate change on the possible future distribution of organisms have been well studied in recent years. For example, using species distribution models (SDMs), Warren et al. [19] estimated the potential effects of climate change on the future distribution of 48,786 species on a global scale. They found that 57% of all plants and 34% of all animals will have an almost 50% change in their future distribution in 2100 under climate change scenarios. However, by studying the literature on mutualist networks such as plants–pollinators, we find that a few studies have been conducted in this field [13,29,30,31]. One of the reasons is that we can estimate the potential effects of climate change on the future distribution of plants and pollinating insects separately, but it is difficult to estimate the effects on the new networks that arise from these changes in different parts of the world. Therefore, researchers usually try to estimate these effects on plant–pollinator networks using simulation models [32].

In one of the first attempts, Devoto et al. [33] simulated species extinction and consequent disruption of the topology of plant–pollinator networks under climate change. In the past 10 years, there have been attempts made to study the impacts of climate change on interconnected networks involving plants and pollinators. For example, Schleuning et al. [25] used SDMs to estimate climate change effects on the future distribution of 295 plants, 196 bees, 70 butterflies, and 97 hoverflies under climate change scenarios. Then, based on eight quantitative matrices of the interactions of plants and pollinating insects, they tried to estimate the effects of climate change on species extinction. The species that were most likely to decrease in the future were removed from the networks sequentially, and then the effect of this removal on the whole network was evaluated. Vizentin-Bugoni et al. [11] also introduced a new approach that integrates the capability of species to substitute lost connections into a prevalent coextension model, aimed at gauging the resilience of networks. However, Vizentin-Bugoni et al. [11] used simulation steps to incorporate rewiring in the estimation of the pollinators’ robustness to plant loss. 

In plant–pollinator networks, ‘rewiring’ refers to the process where species form new interactions with different partners in response to changes in environmental conditions or species composition. This occurs when a species loses its current pollinators or plant partners and connects with alternative species that can perform similar ecological roles. Rewiring is vital for the resilience and stability of ecological networks, enabling species to preserve functional relationships and adapt to changing environments, such as those caused by climate change [34,35]. Previous studies [11,36] have been limited in their assessment of climate change impacts on plant–pollinator networks by relying on indirect estimations through assumptions and simulations. These approaches often led to unrealistic estimations, hindering the ability to draw meaningful global conclusions about these effects. To address this, we aim to introduce a novel approach for gauging climate change effects on plant–pollinator networks, offering a more pragmatic and realistic estimation compared to prior research endeavors. 

Our approach primarily addresses an indirect consequence of spatial mismatch due to climate change impacting plants and pollinators, specifically focusing on spatial mismatch. While it is feasible to calculate spatial discrepancies using SDMs for individual species pairs [37], there is a need to gauge these impacts within a mutualistic network context. Through this approach, we incorporate genuine discrepancies between plants and pollinators, as opposed to the simulated approaches used in earlier studies. Our approach provides insights into how network rewiring might mitigate or intensify the effects of climate change on mutualistic interactions. Unlike joint species distribution models (JSDMs), which account for species interactions and co-occurrence, our method relies on species distribution models (SDMs) to focus specifically on individual species distributions based on environmental variables [38]. This enables us to analyze how particular environmental factors affect the presence or absence of species independently, without considering their interactions with other species.

## 2. Method Description 

Our approach relies on SDMs; hence, to highlight the importance and practical application of SDMs in studying the effects of climate change on plant–pollinator interactions, we first offer an overview of SDMs and deal with their outputs. Subsequently, we demonstrate how these outputs, in the form of distribution maps for plants and pollinators, can be overlapped. For each location or cell within the study area, this overlapping yields a matrix outlining potential connections between plants and pollinators, implicitly considering the rewiring process. We then introduce a Python program developed for this purpose, showcasing its ease of use and simplicity. We aim to facilitate users in seamlessly integrating our newly introduced model into their research endeavors.

### 2.1. SDMs 

SDMs, also referred to as ecological niche models (ENMs) or habitat selection models, find extensive application in ecology, evolutionary biology, and conservation [39,40,41,42,43]. SDMs offer insights into both commonalities and peculiarities regarding the factors influencing the intricate spatial distributions of species. SDMs are numerical tools that combine species presence points with environmental factors. The central idea of SDMs is the niche theory that was introduced by Joseph Grinnell and G. Evelyn Hutchinson [43,44,45]. As input, SDMs require georeferenced individual locations or species’ presence as the response or dependent variable, and independent layers of environmental information such as climate, slope, elevation, land cover, and soil attributes. In numerous studies, SDMs have also been employed to assess the impact of climate change on both plants and insects [46,47,48,49,50,51,52]. 

### 2.2. Outputs of SDMs

The outcome generated by SDM models typically manifests as a raster map, where each cell denotes a numerical value signifying the likelihood of a species’ presence or the suitability of the habitat for that species. These values usually range between 0 and 1, constituting a continuous spectrum. However, in certain scenarios, it becomes necessary to categorize this map into two classes: 0 and 1, representing the absence and presence of the species, respectively. Consequently, a standard for this classification must be established, tailored to the study’s objectives and the specific characteristics of the species under investigation. Numerous studies have tackled this issue, offering recommendations to enhance the conversion of continuous maps into binary representations [53,54,55,56,57].

**Figure 1:**
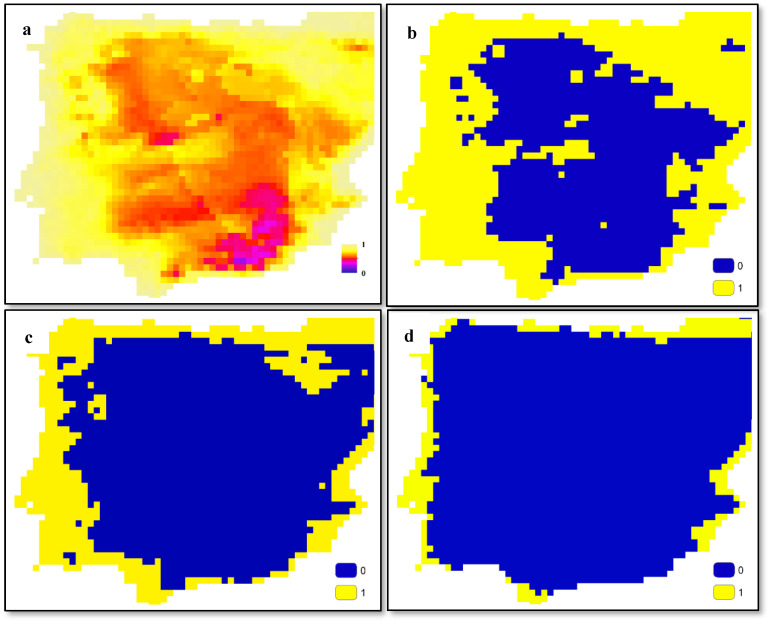
Examples of SDM output (**a**) and threshold-based classified maps for values 0.6 (**b**), 0.7 (**c**), and 0.8 (**d**).

To illustrate the outputs of SDMs more effectively, Figure 1 presents a continuous map (Figure 1a) derived from SDM outputs, which has been transformed into binary maps using three threshold values: 0.6 (Figure 1b), 0.7 (Figure 1c), and 0.8 (Figure 1d). Adjusting these threshold values allows us to manipulate which cells are considered to represent the presence of the species, potentially excluding cells where the species is present from our analyses by increasing the threshold values. These maps can be interpreted as the distribution maps of either a plant or a pollinator within a specific area. Regardless of the species being studied, the typical output of SDMs resembles these maps. In the methodology outlined in this study, it is necessary to convert these continuous maps into binary maps.

### 2.3. Overlapping of Outputs of SDMs

**Figure 2:**
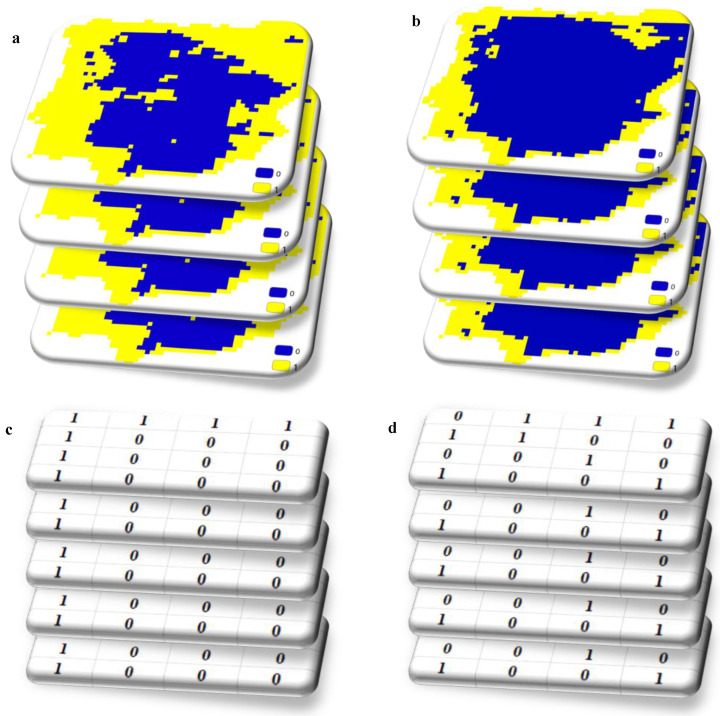
Schematic representation of overlapping binary suitability maps of plants (**a**,**c**) and pollinators (**b**,**d**).

Let us imagine we have gathered presence data for various plants and their associated pollinators, creating distribution maps based on certain environmental factors. These maps outline where these species are likely to thrive. After converting these suitability maps into binary maps to delineate their geographical ranges, we end up with numerous binary maps for both plants and pollinators. Figure 2 illustrates the process of creating these binary maps. By overlaying the binary maps of plants and pollinators, we essentially stack these matrices consisting of 0s and 1s on top of each other. For instance, in a particular geographic location, if there are 5 plants and 5 pollinators, we would have 10 values in one cell. Some of these values may be 0, indicating absence, while others may be 1, indicating presence.

### 2.4. Interaction Network Extraction from Binary Maps

We can consider a cell in the pollinator map to show a ‘1’, indicating the presence of a pollinating insect in that specific area. When a cell in the pollinator map has a ‘1’ (present), we can check all the plants present (cell value of 1) in that same cell to see if they potentially interact with this pollinator. We can repeat this process for every cell, effectively generating networks of interactions between plants and pollinators. To clarify, “If both a plant and a pollinator share a cell with values of ‘1’, we consider them as interacting and mark this in our network. If their cells do not overlap (i.e., one or both have a value of ‘0’), we assume no interaction between them, as neither is present in the same suitable area”.

It is important to highlight the process involved in extracting interaction matrices between plants and pollinators. The dimensions of this matrix are solely determined by the number of plant and pollinator maps. For instance, if we have generated maps for 100 plants and 100 pollinators, we would have one matrix per cell with dimensions of 100 × 100, populated with 1s and 0s to denote the presence or absence of interactions. Typically, the column headings contain the names of pollinators, while plant names are listed in the rows. Therefore, a software or program capable of aggregating these maps, distinguishing between plant and pollinator maps, organizing species names accurately, and assigning appropriate interaction values based on species–species co-overlap is essential. Given the complexity of these tasks, we opted for the Python programming language to develop such a program. 

**Figure 3:**
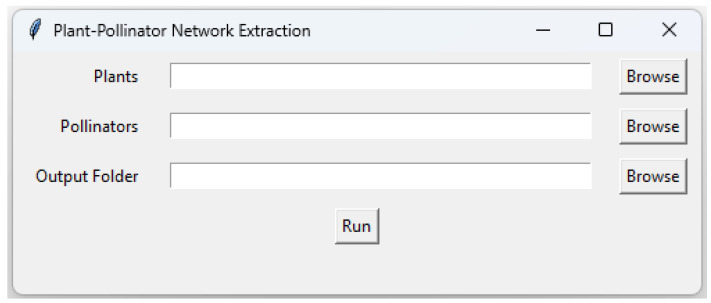
Graphical user interface (GUI) of the Python program for extracting interaction networks of plants and pollinators.

For this, we developed a Python program that requires two folders containing binary distribution maps for plants and pollinators separately. It automatically extracts the names of plants and pollinators and creates matrices with rows representing plants and columns representing pollinators. These matrices capture potential interactions by assigning ‘0’ where there is no overlap between a plant and a pollinator and ‘1’ where their ‘1’ cells coincide. The code generates separate CSV files, each representing a matrix for every cell, enabling users to review and remove files without any interactions between plants and pollinators. This program has a user-friendly graphical interface (Figure 3) to facilitate its usage. The program’s code (Python 3.12) is available as a TXT file on GitHub (https://github.com/ehsanrahimi666/SDM.git, accessed on 1 October 2024), allowing users to review and modify it as needed. To utilize the program, users simply need to copy the code into a Python script and execute it. 

Upon execution, the program will launch a graphical interface prompting users to input three paths: one for the binary maps of plants and pollinators, and another for the output path where the matrices will be saved, with each cell of the specified area represented. The naming convention for these matrices is based on latitude and longitude coordinates, making it advisable for users to utilize the WGS 84 geographic coordinate system for their maps. To ensure our networks reflect actual interactions, we need to refer to an established interaction matrix or reference matrix. This matrix helps refine our networks, removing any extra links that do not align with known interactions. 

### 2.5. Estimating Climate Change Effects on Plant–Pollinator Interactions

In the context of SDMs, climate change scenarios such as the Shared Socioeconomic Pathways (SSPs) [58,59] serve as essential inputs for projecting the future distribution of pollinators [21]. By coupling climate projections derived from these scenarios with species occurrence data and environmental variables, researchers can develop models that estimate how species distributions may shift in response to changing climatic conditions. For instance, researchers can use downscaled climate data from global circulation models (GCMs) under different SSP scenarios to generate future climate projections at finer spatial scales. These climate projections can then be incorporated into SDMs to simulate how species habitats may expand, contract, or shift geographically under different climate change scenarios. By simulating various climate change scenarios using SDMs, researchers can assess the potential range shifts, habitat suitability changes, and extinction risks for different species under different future climate conditions. 

Under different scenarios of climate change, we still need to use SDMs whose output was described in detail earlier. Therefore, to investigate the effects of climate change on the interactions between plants and pollinators, the matrix extraction process that was explained before is performed once for the present time and again for the binary maps that will be created for the same species in future scenarios. Therefore, in this case, for each cell in the study area, we will have a matrix for the current scenario and a matrix for future scenarios, and by comparing these two matrices, we can find out which species have been generally removed from the target cell or lost their connections with other species. Additionally, in this situation, the rewiring process makes the pollinators that interacted with other plants in that cell before, under the climate change scenario, possibly interact with new plants that may migrate to this cell. 

### 2.6. Application to a Case Study

#### 2.6.1. Plant–Pollinator Catalog for Chile

To implement our methodology, which integrates rewiring and its implications in climate change effects, it is essential to have a dependable dataset documenting interactions between plants and pollinators. Consequently, one such reliable resource detailing interactions between plants and pollinators in Chile has been available by Muschett et al. [60]. They have gathered and synthesized data sourced from published scientific literature about pollinators, flower visitors, and interactions between plants and pollinators in Chile. Their compilation encompasses 120 publications, yielding a total of 2619 records. These records provide details on the location, habitat type, and methods of establishment for 357 plant species across 83 families (see Supplementary Materials). Subsequently, they constructed a comprehensive database consolidating information regarding their pollinators and flower visitors, encompassing data on 492 pollinator species originating from 97 families and spanning 13 orders. Hence, this catalog served as the foundational dataset for our research endeavor. 

#### 2.6.2. Occurrence Data

The Chile catalog initially listed 357 plant species and 492 pollinator species. However, it lacked occurrence data points for the species included. As a result, we had to search the Global Biodiversity Information Facility (GBIF) website (www.gbif.org, accessed on 1 January 2024) to locate this type of data for both plants and pollinators. Even with this effort, all species may not have sufficient presence points available. The number of occurrence data points required for species distribution modeling (SDM) depends on various factors such as the complexity of the model, the quality of the data, species characteristics, research goals, and the scale of the study [61]. While there is not a strict rule, larger sample sizes are generally preferred as they offer more detailed information for accurate modeling. A commonly suggested guideline is to aim for at least 30 to 50 presence points, though this can vary depending on the specific circumstances [62,63]. In our study, we set the minimum requirement at 30 data points in Chile (see Supplementary Materials). 

This process resulted in a refined selection, yielding 187 plant species and 171 pollinators or visitors for our analysis. To maintain data accuracy, we conducted a detailed review of the presence points to eliminate duplicate entries and those falling outside the study area. The “rgbif” v3.8.0 package, an easy-to-use interface in R v4.2.3 for accessing biodiversity data from GBIF, was crucial for data processing. To improve the accuracy of our occurrence data, we applied a 10 km distance filter. This means that for both plants and pollinators, we excluded any duplicate records within a 10 km radius, retaining only one occurrence point per species in these overlapping areas. This helps reduce the spatial clustering or over-representation of species in certain locations, ensuring that the occurrence data better reflect the broader distribution of each species.

#### 2.6.3. Environmental Variables

Climate is a key factor in determining the ecological niche of species, as organisms are highly adapted to their local climate conditions. Changes in these conditions can affect their typical behaviors, such as feeding and mating. Factors such as temperature, precipitation, and solar radiation directly impact these activities [64,65]. Terrestrial species, in particular, thrive within specific climate conditions, known as their climate niche, which is defined by certain temperature and precipitation ranges. Several studies have highlighted the importance of climate variables in shaping species distribution [66,67,68,69,70,71,72,73,74]. In our study, we obtained predictor variables from the WorldClim database (www.worldclim.org, accessed on 1 January 2024), specifically bioclimatic layers consisting of 11 temperature and 8 precipitation variables. As these variables often exhibit high correlations, it is cautioned against using the complete set in species distribution modeling. 

Therefore, employing the usdm [75] package, we conducted a step-wise process based on Variance Inflation Factor (VIF) to exclude highly correlated variables. The resultant variables include the Mean Diurnal Range (Bio 2), Isothermality (Bio 3), Temperature Seasonality (Bio 4), Mean Temperature of Wettest Quarter (Bio 8), Precipitation of Wettest Month (Bio 13), Precipitation of Driest Month (Bio 14), Precipitation Seasonality (Bio 15), Precipitation of Warmest Quarter (Bio 18), and Precipitation of Coldest Quarter (Bio 19). Furthermore, we utilized the SSP585 climate change scenarios—a trajectory associated with a substantial increase in CO_2_ emissions and a temperature surge to 4.4 by 2070—to model the prospective distribution of plants and pollinators for the year 2070. SSP585 is recognized as one of the most extreme scenarios for climate change [76].

#### 2.6.4. Model Fitting

In our research, we employed the “FLEXSDM” R package [77] to model the distribution of the species under investigation. Utilizing the MaxEnt model, we generated climate suitability maps by integrating presence data with climate information. To facilitate the modeling process, we created 5000 random pseudo-absence points, which were utilized alongside the presence points in Chile within the MaxEnt model. In this study, we categorized the climate suitability map into two classes: low and high suitability. The high-suitability class encompassed values exceeding 0.6 [78] or 0.7 [21,61]. Therefore, the threshold in this study was selected based on expert opinion, with guidance from previous studies. We focused our analysis solely on the range changes within the high-suitability class. To calculate the percentage of change, we employed the following formula:

Percentage of change = ((number of cells in the future classified as high suitability) − (number of cells in the current classified as high suitability))/(number of cells in the current classified as high suitability)) * 100

This formula allowed us to quantify the percentage change in high-suitability areas between the present and future conditions.

#### 2.6.5. Model Assessment Results

To assess the performance of the different models, we employed three evaluation metrics: inverse mean absolute error (IMAE), the area under the ROC curve (AUC), and the Boyce Statistic (BOYCE) utilizing the “FLEXSDM” R package [77]. AUC values typically ranging between 0.7 and 0.9 are considered acceptable, and values surpassing 0.9 denote excellent results, indicating highly accurate model predictions. IMAE is calculated as 1— (Mean Absolute Error), aligning with other metrics where higher values signify greater model accuracy. The Boyce Index evaluates the predictive performance of species distribution models (SDMs) by measuring the concordance between the observed and predicted probabilities across different predicted suitability levels. Its values range from −1 to 1, where approaching 1 suggests a strong alignment between the observed and predicted probabilities, signifying the model’s effectiveness across varied probability thresholds. Values near 0 indicate a performance similar to random chance, while values below 0 indicate a performance that is worse than random predictions. To evaluate these metrics consistently, we employed 5-fold cross-validation [79] as part of the model evaluation process.

#### 2.6.6. Bipartite Metrics Results

To assess various attributes of the extracted networks, we utilized the bipartite package [80,81] within the R software. For each network derived from raster cells at the network level, we computed 12 metrics (Table 1). The network metrics provide an overview of the entire network and offer insights into higher and lower trophic levels. The suffixes LL and HL denote lower and higher levels, respectively. A detailed description of each metric is outlined in Table 1. 

## 3. Results

### 3.1. Model Assessment

Table 2 shows the results of model validation metrics for different groups based on the AUC, BOYCE, and IMAE statistics. According to this table, the AUC values for plants and pollinators are 0.99 and 0.98, respectively, which indicates that the results of the models are excellent. According to the other statistics, our predictions also fall within the excellent range. 

### 3.2. Potential Effects of Climate Change at the Individual Level

**Figure 4:**
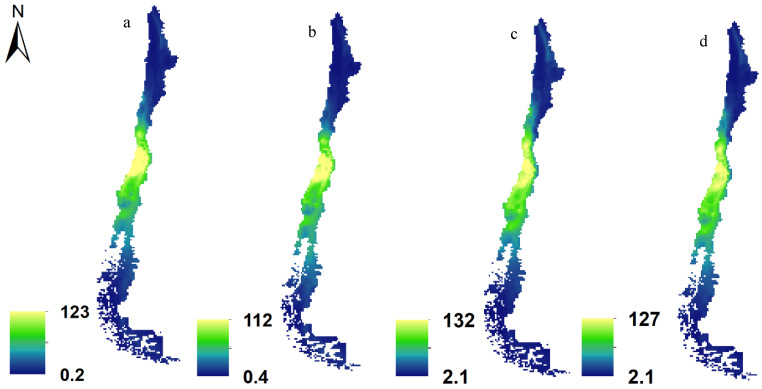
Richness maps of plants (**a**,**b**) and pollinators (**c**,**d**) in current (**a**,**c**) and future (**b**,**d**) (SSP585, 2070) scenarios in Chile (cell size = 1 km^2^).

Figure 4 depicts plants and pollinators in current (Figure 4a,c) and future (Figure 4b,d) (SSP585, 2070) scenarios. In preparing these maps, we summed the distribution maps for 187 plants and 171 pollinators across two time frames: the present and the future. The figure highlights that the highest habitat suitability for both pollinators and plants is concentrated in the central regions of Chile specifically around the nation’s capital, Santiago. However, due to the overlay of numerous maps, it becomes challenging to distinctly observe changes in species distribution through this visual representation. Therefore, we offer a more detailed account of the distribution changes in plants and pollinators in Table 3. Table 3 presents the average percentage changes in areas classified as highly suitable under the climate change scenario projected for Chile in 2070. The “No. species” column reflects the number of species expected to experience shifts in their distribution ranges due to climate change, indicating whether these changes involve an expansion or contraction. The values in parentheses represent the respective standard deviations.

For plants, it is projected that 40 species will experience an increase in their distribution range by an average of 18.3%, with a standard deviation of 14. In contrast, 147 plant species are expected to see a decrease in distribution by an average of 33.4%. Regarding pollinators, this analysis suggests that 47 species may witness an increase in distribution by an average of 24.3%. Conversely, 124 pollinator species are projected to experience a decrease in distribution by an average of 25.7%. 

### 3.3. Potential Effects of Climate Change at the Network Level

**Figure 5:**
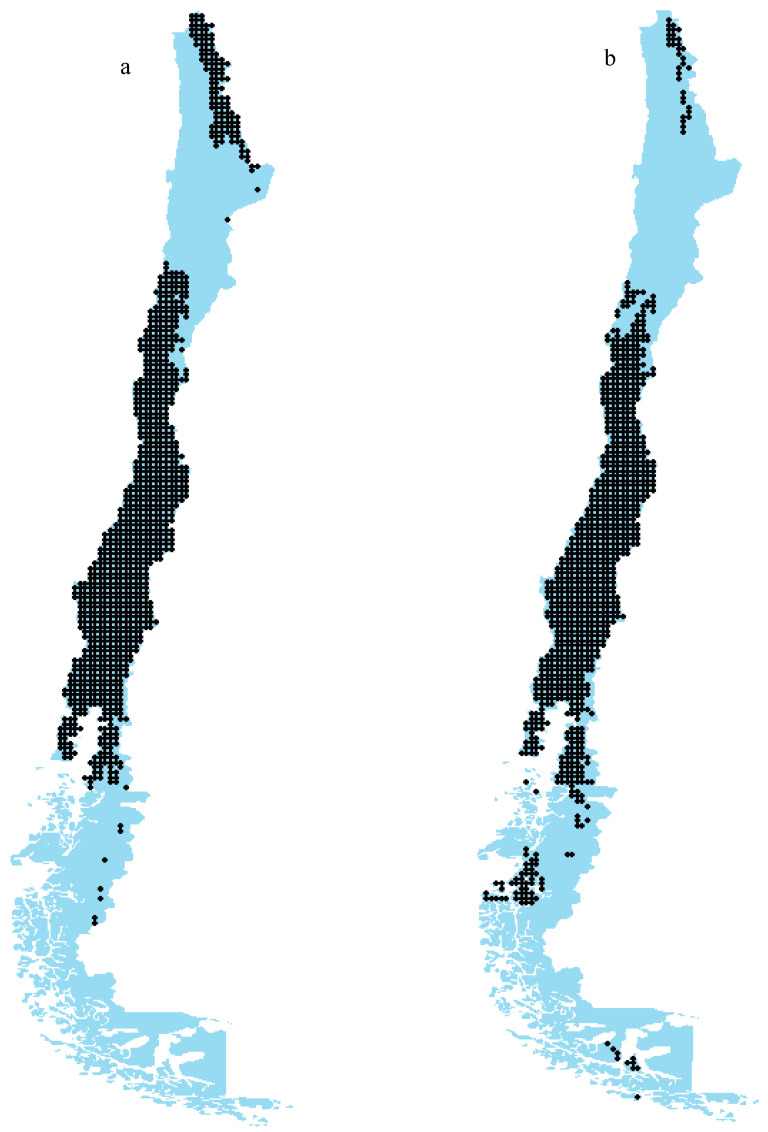
Network location of plant–pollinator networks in current (**a**) and future (**b**) (SSP585, 2070) scenarios in Chile (cell size = 1 km^2^). The blue color indicates regions lacking networks, while each black dot represents a network.

Figure 5 illustrates the spatial distribution of the extracted plant–pollinator networks in the current (Figure 5a) and future (Figure 5b) scenarios in Chile, emphasizing their prevalence in the central regions. Notably, in the present scenario, the northern regions of Chile host numerous locations with established networks, whereas the southern regions exhibit a limited presence of such networks. However, in the future scenario, a notable shift occurs: the number of networks in the northern areas declines, while the southern regions experience an increase in network occurrences. This may suggest a significant redistribution of plant–pollinator interactions in response to changing climatic conditions. This study encompassed a total of 2906 cells (maximum potential networks) throughout Chile, resulting in plant–pollinator networks for 1136 cells in the present and 1036 cells in the future. The decline in network formation is attributed to the loss of species due to climate change, diminishing the potential for reciprocal interactions between these species.

### 3.4. Bipartite Metrics

Table 4 presents network-level bipartite metrics for both the current and future scenarios, with the suffixes LL and HL denoting lower and higher levels, respectively. The values presented in this table denote the average values derived from the overall metrics computed for the networks. It is important to highlight that the bipartite package may not apply to extremely small networks, such as those with just one connection between a plant and a pollinator, preventing the calculation of certain indicators. Consequently, the average values in this table are based solely on the metrics that could be computed for the networks under consideration. Connectance, representing the proportion of realized links to all possible links, increases from 0.25 to 0.31 in the future on average. Web asymmetry, reflecting the asymmetrical distribution of links between plants and pollinators, sees a minor decrease from −0.18 to −0.19. Links per species decrease slightly from 1.13 to 1.05. Modularity Q, indicating the degree of compartmentalization within the network, shows a marginal increase from 0.53 to 0.55. Nestedness, NODF, and Weighted nestedness, which measure the ordered arrangement of interactions, decrease slightly in the future. Linkage density, indicating the average number of links per species, decreases from 5.91 to 5.29. Robustness generally maintains the same values between current and future scenarios.

## 4. Discussion

Our analysis of the impact of climate change on individual plant and pollinator species in Chile reveals a notable trend toward a decreased future distribution in 2070. Approximately 75% of the 358 species studied are anticipated to undergo a reduction in distribution, with an estimated decrease of around −25% for pollinators and about −33% for plants. These individual-level findings highlight the substantial alterations in the distribution patterns of both plants and pollinators, raising concerns about the potential ecological consequences. Consistent with expectations, the anticipated substantial impacts of climate change on spatial mismatches at the individual level are not evident in the network-scale analysis. While it is predicted that 100 cells or locations in Chile may lack plant–pollinator networks in the future under the climate change scenario, other intensified effects of climate change do not manifest in existing networks.

Notably, Table 4 indicates minimal discernible differences in metrics between the present and future, despite a projected decrease in 75% of species. Specifically, metrics such as robustness did not show any change due to climate change. This could be attributed to two factors: firstly, the decline in plant and pollinator numbers may not be sufficient to induce a significant change in network topology, and secondly, bipartite metrics may exhibit low sensitivity to small alterations in networks, making it challenging to detect subtle changes appreciably. Plant–pollinator networks are markedly more vulnerable to the loss of generalist animals and plants than to random species loss or the loss of species with few interactions [11,82], or are more sensitive to plants than to animal extinction under climate change [25]. Nonetheless, in this study, we did not delve into an in-depth analysis of whether there will be a substantial decrease in the distribution of generalist or specialist species. It is believed that the loss of generalists may impact network cohesiveness by connecting distinct network modules, influencing network stability and resilience to disturbances [83]. However, the presence of constraints on interactions may prevent significant declines in extinction rates. This relatively resilient response to extinction is then attributed to redundancy in pollinators per plant and the nested topology of the networks [84]. 

For example, Memmott et al. [82] investigated potential extinction patterns in two extensive networks involving plants and their flower visitors by simulating the elimination of pollinators, leading to the subsequent loss of plants dependent on them for reproduction. In each network, they conducted removal simulations in three ways: randomly, systematically from the least-linked (most specialized) to the most-linked (most generalized), and systematically from the most-linked to the least-linked pollinators. The decline in plant species diversity was most rapid when removing the most-linked pollinators, but the declines did not exceed a linear pattern. Networks characterized by distinct, densely connected subsystems are termed modular [85]. In ecological contexts, there has also been a hypothesis suggesting that a modular structure in species interactions could enhance the dynamic stability of communities, although supporting evidence for the stability of mutualistic networks is found in this study. Modularity can exert moderate stabilizing effects under specific parameter conditions, while anti-modularity has the potential to significantly destabilize ecological networks [85].

## 5. Study Limitations

Any new study or model developed to solve a problem may come with certain limitations. Our proposed method is no exception, and here, we outline its limitations while identifying the conditions under which it can be most effective. The primary limitation of our method is its reliance on SDMs, meaning that all assumptions typically made when using SDMs also apply to our approach. These assumptions include species–environment equilibrium, absence of observation bias, independence of species observations, availability of all key predictors, and error-free predictors, among others [44,86]. Therefore, the limitations inherent to SDMs directly affect the outcomes of our method, as it is fully based on SDMs.

Another limitation is the uncertainty associated with climate change scenarios for the period between 2030 and 2100 [87]. These scenarios range from optimistic to pessimistic, and their inherent uncertainty can significantly influence our method’s results. To address this, it is advisable not to rely on a single scenario but rather to use multiple scenarios to assess the sensitivity of the method. Additionally, our method uses binary maps, which require users to convert species distribution data into binary form. We used the threshold of 0.6 in this study; however, different thresholds such as 0.9 or 0.3 could lead to significant changes in the results. To minimize sensitivity, it is recommended to create binary maps under different scenarios. Another important factor is the potential bias in presence points or the lack of access to accurate data for a given area [88]. This limitation can prevent the application of SDMs to certain species and regions. All the limitations of SDMs are not specific to our method but rather general criticisms of SDMs. However, thousands of studies have successfully used these models, and researchers have worked to reduce these limitations to produce more reliable results [89]. If users of our model similarly address these limitations, they can improve the reliability of their findings.

Our method also includes a second component focused on plants and pollinators. To estimate the effects of climate change on plant–pollinator networks, it is essential to ensure that the study area has been adequately sampled and that the geographic locations of each species’ presence are recorded. This allows us to provide the necessary presence points for modeling in advance. Otherwise, users may have to rely on databases such as GBIF, which come with their limitations. For example, while Chile could serve as an ideal study area due to extensive research, our method can only be applied to the 492 pollinator species and 357 plant species recorded, despite the country hosting thousands of species. Chile is one of the few regions where ecologists have documented plant–pollinator relationships in around 120 studies [61]. However, since the geographic locations of many species were not recorded, we had to rely on GBIF data. Unfortunately, presence points were only available for 187 plant species and 171 pollinators. Thus, insufficient sampling and incomplete data on species presence can affect the outcomes of our method. These limitations do not represent flaws in our method. Our approach can produce reliable results, provided users have adequate data and key presence points when applying species distribution models.

## 6. Conclusions

This study offered a comprehensive assessment of the implications of climate change on plant and pollinator interactions in Chile, utilizing a novel methodology that bridges existing research gaps. By integrating species distribution models with interaction matrices, our approach facilitates a more holistic understanding of how climate change influences both individual species distributions and their interconnections within ecological networks. This analysis revealed a concerning trend: a significant proportion of species are expected to face reduced distributions, emphasizing the potential ecological consequences of climate change. Our results indicated that network extinctions may be the predominant consequence of climate change, rather than substantial alterations in network topology. As the ecological landscape continues to evolve, ongoing research will be essential to refine these models and develop effective conservation strategies aimed at mitigating the impacts of climate change on plant–pollinator networks.

## Figures and Tables

**Table 1:** Network-level bipartite metrics and their definition. The suffixes LL and HL refer to lower and higher levels, respectively.

Metric	Definition
Connectance	The realized proportion of possible links
Web asymmetry	The balance between numbers in the two levels: positive values indicate higher trophic level species, negative values indicate more lower-trophic-level species
Links per species	The mean number of links per species (qualitative): sum of links divided by number of species
Modularity Q	Compartments are sub-sets of the web that are not connected (through either higher or lower trophic levels) to another compartment
Nestedness	The nestedness temperature of the matrix (0 means cold, i.e., high nestedness, 100 means hot, i.e., chaos)
NODF	Another index for nestedness: high values indicate nestedness.
Weighted nestedness	A nestedness version that considers interaction frequencies (and is hence weighted)
Linkage density	Marginal total-weighted diversity of interactions per species (quantitative)
Number of species HL	Number of pollinators
Number of species LL	Number of plants
Robustness HL	The area below the “secondary extinction” curve for pollinators
Robustness LL	The area below the “secondary extinction” curve for plants

**Table 2:** Model validation metrics including AUC, BOYCE, and IMAE generated using the MaxEnt algorithm for bees. The standard deviations are presented in parentheses.

Contingent	AUC	BOYCE	IMAE
Plants	0.99 (0.00)	0.93 (0.04)	0.98 (0.01)
Pollinators	0.98 (0.01)	0.92 (0.05)	0.97 (0.01)

**Table 3:** The average percentage of changes in the area of the high-suitability class under climate change scenario in 2070 in Chile. The No. Species column (increase/decrease) represents the number of species whose distribution range will change under climate change scenarios. The numbers in parentheses also show the standard deviations.

	No. Species (Increase)	Increase%	No. Species (Decrease)	Decrease%
Plants	40	18.3 (14)	147	−33.4 (20)
Pollinators	47	24.3 (32)	124	−25.7 (18)

**Table 4:** Network-level bipartite metrics and the average values derived from the overall metrics computed for the networks (n = 1136 networks for current scenario, and 1036 networks for future scenario).

Bipartite Metrics	Current	Future
Connectance	0.25	0.31
Web asymmetry	−0.18	−0.19
Links per species	1.13	1.05
Modularity Q	0.53	0.55
Nestedness	13.48	12.99
NODF	23.77	24.09
Weighted nestedness	0.64	0.67
Linkage density	5.91	5.29
Number of species HL	25.55	19.29
Number of species LL	33.22	27.35
Robustness HL	0.49	0.49
Robustness LL	0.55	0.55

## Data Availability

The Python program and example raster binaries of plants and pollinators for Chile presented in this study are openly available at https://github.com/ehsanrahimi666/SDM.git, accessed on 1 October 2024.

## Supplementary Materials

The following supporting information can be downloaded at: https://github.com/ehsanrahimi666/SDM.git, accessed on 1 October 2024.
